# Genetic Diversity and Low Stratification of the Population of the United Arab Emirates

**DOI:** 10.3389/fgene.2020.00608

**Published:** 2020-06-12

**Authors:** Guan K. Tay, Andreas Henschel, Gihan Daw Elbait, Habiba S. Al Safar

**Affiliations:** ^1^Center for Biotechnology, Khalifa University of Science and Technology, Abu Dhabi, United Arab Emirates; ^2^Department of Biomedical Engineering, Khalifa University of Science and Technology, Abu Dhabi, United Arab Emirates; ^3^Faculty of Health and Medical Sciences, UWA Medical School, The University of Western Australia, Crawley, WA, Australia; ^4^School of Medical and Health Sciences, Edith Cowan University, Joondalup, WA, Australia; ^5^Department of Computer Science, Khalifa University of Science and Technology, Abu Dhabi, United Arab Emirates; ^6^Department of Genetics and Molecular Biology, College of Medicine and Health Sciences, Khalifa University of Science and Technology, Abu Dhabi, United Arab Emirates

**Keywords:** genetic anthropology, population genetic variation, next generation sequencing, population admixture, population-specific allele frequencies

## Abstract

With high consanguinity rates on the Arabian Peninsula, it would not have been unexpected if the population of the United Arab Emirates (UAE) was shown to be relatively homogenous. However, this study of 1000 UAE nationals provided a contrasting perspective, one of a relatively heterogeneous population. Located at the apex of Europe, Asia, and Africa, the observed diversity could be explained by a plethora of migration patterns since the first Out-of-Africa movement. A strategy to explore the extent of genetic variation of the population of the UAE is presented. The first step involved a comprehensive population stratification study that was instructive for subsequent whole genome sequencing (WGS) of suitable representatives (which is described elsewhere). When these UAE data were compared to previous smaller studies from the region, the findings were consistent with a population that is a diverse and admixed group of people. However, rather than sharp and distinctive clusters, cluster analysis reveals low levels of stratification throughout the population. UAE emirates exhibit high within-Emirate-distance/among-Emirate distance ratios. Supervised admixture analysis showed a continuous gradient of ancestral populations, suggesting that admixture on the south eastern tip of the Arabian Peninsula occurred gradually. When visualized using a unique technique that combined admixture ratios and principal component analysis (PCA), unappreciated diversity was revealed while mitigating projection bias of conventional PCA. We observe low population stratification in the UAE in terms of homozygosity versus separation cluster coefficients. This holds for the UAE in a global context as well as for isolated cluster analysis of the Emirati birthplaces. However, the subtle clustering observed in the Emirates reflects geographic proximity and historic migration events. The analytical strategy used here highlights the complementary nature of data from genotype array and WGS for anthropological studies. Specifically, genotype array data were instructive to select representative subjects for WGS. Furthermore, from the 2.3 million allele frequencies obtained from genotype arrays, we identified 46,481 loci with allele frequencies that were significantly different with respect to other world populations. This comparison of allele frequencies facilitates variant prioritization in common diseases. In addition, these loci bear great potential as biomarkers in anthropological and forensic studies.

## Introduction

Comprehensive reference panels of genetic variation for entire populations are paramount for the interpretation of the medical and functional relevance of DNA sequence changes. The Arabian peninsula is notoriously understudied with only 0.08% of human genetic sequences in public databases from populations of a region (Popejoy and Fullerton, 2016). This study was commissioned since all genomes matter, irrespective of ethnic background. No whole genome sequencing (WGS) datasets in the public domain to date are from the people of the United Arab Emirates (UAE), except for (AlSafar et al., 2019). There are notable sequencing efforts in the region from Qatar (Omberg et al., 2012; Fakhro et al., 2016) and Kuwait (Alsmadi et al., 2013, 2014; John et al., 2015; Thareja et al., 2015). Nevertheless, there are differences between populations studied in these two genome efforts when compared to this UAE effort.

The UAE is located in the south eastern corner of the Arabian peninsula. Apart from the UAE, the Arabian Peninsula comprises Kuwait, Bahrain, Oman, Qatar, Saudi Arabia, Yemen, as well as parts of Iraq and Jordan. It is an ethnically diverse area that has arisen from a series of social and cultural influences. Collectively, the inhabitants are referred to as “Arabs”; a major pan-ethnic group that inhabits Western Asia, North Africa, and parts of the Horn of Africa. Arabs are so defined as they share common linguistic, cultural, religious, and political traditions. Countries such as Kuwait and Saudi Arabia have land borders with Asian counterparts, hence are influenced by overland trade routes from the east, and the north, respectively. In contrast, the UAE in the south east is relatively isolated with land borders with Saudi Arabia and Oman. Migration from Asia over the relatively short distance that is the Straits of Hormuz was convenient and did occur. The efforts described here provide genetic information that complements and adds to the body of knowledge relating to immigration, providing an understanding of the peopling of the south east region of the Arabian peninsula.

The Arabian peninsula sits at the crossroads of significant human migration between the African, European, and Asian continents. Mitochondrial DNA (mtDNA) analyses have been widely used as a tool for human migration studies (Kundu and Ghosh, 2015). Archeological evidence, as well as human genetic diversity and phylogenetic analyses, suggest that peopling of the world originated in Africa (Cann et al., 1987; Nielsen et al., 2017). With the use of mtDNA analysis, the first wave of modern human migration out of Africa has been postulated to have occurred approximately 85,000 years ago (Cann et al., 1987; Forster, 2004; Kundu and Ghosh, 2015). On the other hand, the earliest archeological evidence of anatomically modern humans on contemporary UAE soil dates back to 127,000 years ago (Armitage et al., 2011) and reconciliation of archeological and genetic evidence remains an open challenge. Two proposed routes of human migration out of Africa and into the Middle East have been postulated. The first and obvious route took place across the land bridge that is now Egypt into the Levantine region (Nielsen et al., 2017). The second “southern” route was from a location within contemporary Djibouti across a relatively shallow stretch of water referred to as Bab al Mandab Strait into what is now Yemen in the South Western corner of the Arabian peninsula (Shepard and Herrera, 2006). Prehistoric hunter-gatherer communities were supported by a more conducive climate and environment (Petraglia and Rose, 2009). However, it is not clear, whether continuous populations occupied the peninsula since its earliest settlement, and whether later immigration waves superseded or interbred with previous ones.

The Neolithic Revolution in Mesopotamia, the productive land around the Tigris and Euphrates rivers, around 10,000 years ago gave rise to milestones in human development, such as the invention of agriculture, writing, mathematics, and astronomy. Mesopotamia’s impact along the eastern coast of the Arabian Gulf is evidenced by numerous settlements (also in the current UAE) that occurred after the last substantial sea level rise around 6000 to 5000 BCE. They exhibit long distance trade with Mesopotamia and the Indus Valley, pottery, animal husbandry, and permanent stone houses (Lambeck, 1996; Rose, 2010). Intriguingly, the spike of settlements in a short time frame and the rapid rise in cultural complexity stands in stark contrast to all prehistoric evidence of hunter-gatherer communities on the Arabian Peninsula. This fuels speculation about the existence of a “Gulf Oasis,” i.e., a potential refuge for modern humans during hyper-arid times (Rose, 2010). There is evidence in the related Umm al Nar culture for the introduction of advances associated with the Bronze age and agriculture (dates and cereals) to the region around 2600 BCE (Frifelt, 1991).

The eventual development of trade routes (Hodgson et al., 2014) in more recent history has increased bi-directional gene flow (Abu-Amero et al., 2007) into and out of the region, creating the contemporary diversity of modern Arabia.

The original inhabitants of the region led a nomadic existence and moved throughout the peninsula, in search of favorable grazing areas (e.g., oases), resulting in settlements that were the crossroads for trade and cultural exchange. At these ports, admixture probably took place between intermingling populations. Historical texts show that the oversea crossing to Central and South Asia across the Arabian gulf was possible and in fact did take place into the south eastern region of the peninsula, which now includes the UAE. In other parts, populations coalesced at overland ports that connected North Africa and Central Asia. In the northern perimeter, populations would have intermingled with Levantines, and in the south west corner, there were exchanges across the Red Sea with Sub-Saharan Africa. Within the peninsula, there were interconnected routes between these settlements, providing opportunities for further diversification. While evidence for the gene flow between Africa, Southern, and Central Asia during prehistoric times is complex and multi-layered including back migrations (Shepard and Herrera, 2006), more recent migrations are well-established. For example, the influence of Persians from Asia on the southern part of the Arabian Peninsula is known to have taken place during the Achaemenid (700 to 330 BC) and Sassanid (226 to 651 AD) empires and lasted for several centuries (Bey, 1996). The immigration of the Azd, a branch of the Qahtanite tribe, from present day Yemen toward the Hajar Mountains in greater Oman is argued as the event corresponding to the arrival of the first Arabs in the region (Bey, 1996).

As mentioned, the influence from Sub-Saharan Africa into the southern regions of the peninsula including the UAE has arisen by crossings from the Horn of Africa into Yemen (Richards et al., 2003). In addition, the region was impacted during the Portuguese dominance of the Indian Ocean trade (Bey, 1996) and in particular, between Oman and Zanzibar in Tanzania, in more contemporary history. Trade relations between these two settlements gave rise to genetic influence from the African continent. The African influence has also extended into present-day India, as evidenced by a study that describes a population in the western part of India that has been substantially influenced by African populations, specifically Bantu-speakers (Narang et al., 2011). Similarly, a recent study (Fernandes et al., 2019) has shown a dominance of sub-Saharan African admixture in the west of the Arabian Peninsula, whereas South Asian and Levantine/European influence was stronger in the east.

Migration patterns are never unidirectional and back migrations are a common trend of human movement throughout history (Luis et al., 2004; Shepard and Herrera, 2006). Being juxtaposed between the African, European, and Asian continents, it is not surprising that the Arabian Peninsula is at a significant crossroad in human migration as it represents the epicenter of multidirectional flow between populations of the three continents. This is coupled with local migration that would have occurred between settlements within the peninsula. The analyses of mtDNA sequences provide evidence for this pattern (Jones et al., 2017). Analysis of mtDNA and Y chromosome haplogroups have shown a large number of lineage groups in the area which originated from what is now modern Iraq, suggesting that this area was a staging point where populations congregated and dispersed (Al-Zahery et al., 2003). Similarly, modern Iran has a diverse array of mtDNA lineages suggesting that the area between Arabia and Asia (i.e., Persia) was a human migration gateway into Asia and Europe (Thareja et al., 2015). Intrigued by the working hypothesis that historical events related to trade, conquest, cultural exchange, the rise of religion and philosophical pursuits, have shaped a diverse admixture group of people, we present the first comprehensive population stratification study of the UAE.

Contemporary UAE was formed by the union of seven emirates or sheikhdoms in 1971 led by Sheikh Zayed bin Sultan Al Nahyan. The Trucial states that formed the UAE were once located on the southern routes of human migration within the peninsula. To understand the establishment of settlements in the UAE, the genome of its nationals has to be sequenced and analyzed. Of the approximate 10 million population of the UAE, some 15% are citizens of the country. The majority of the residents of the UAE are expatriates, with approximately 30% being South Asian in origin.

With the UAE joining the genome fraternity relatively recently, this study describes the results of a genome analysis pipeline that was optimized to meet our needs, using gene array data to guide the selection of individuals for WGS to study the UAE population.

## Materials and Methods

### Sampling, DNA Extraction, and Genotyping

Prior to enrollment, the 1000 participants provided their written informed consent that had been approved by the Institutional Ethics Committee of Mafraq Hospital in Abu Dhabi, UAE. Subjects were also given a questionnaire to collect their historical and demographical information. The inclusion criteria for the study were that the subjects had to be an adult (>18 years old) citizen of the UAE who understood their contribution to the study and was subsequently able to give consent.

Saliva samples were collected from the participants using the Oragene OGR 500 kit (DNA Genotek, Ottawa, Canada). The prepIT^®^L2P system (DNA Genotek, Ottawa, Canada) was used to extract genomic DNA from buccal cells in the saliva samples. The extracted DNA aliquots were quantified using the DS 11 FX Fluorometer (Denovix Inc., Wilmington, DE, United States) and the integrity of each was checked by electrophoresis on an agarose gel.

### Library Preparation

The samples were genotyped on the Illumina Omni5 Exome Bead Chip v.4.0.1 (Illumina Inc., San Diego, CA, United States) which contains 4.6 million single nucleotide polymorphism (SNP). Illumina recommended the standard protocol was used for sample hybridization and scanning using the Illumina iScan platform.

### Genotype Array Data Pre-processing and Merging of Several Genotyping Datasets

The genotyping data were collected on separate arrays, with 512 (dataset A) and 488 (dataset B) samples yielding dataset AB. The data were merged using PLINK v1.90 (Purcell et al., 2007). For most analyses, we retain only those samples with available birth place in the UAE (758 individuals). The merged dataset was subjected to quality control and then pre-processed according to best practices using PLINK, i.e., we detect SNPs in Linkage Disequilibrium within a window size of 1000, using a step size of 10 and *r*^2^ threshold of 0.4 (plink parameters –indep-pairwise 1000 10 0.4). In addition, we exclude SNPs that fail the Hardy Weinberg Equilibrium test with a significance of 0.001, minor allele frequency < 1%, and missingness > 1%. This step retained 2.3 Million quality controlled loci. In a similar fashion, set AB was merged with 1043 samples from the Human Genome Diversity Project (HGDP) (Cavalli-Sforza, 2005) on the base of intersecting SNPs. This yielded the dataset ABH with 235,478 SNPs for all samples, out of which 35 SNPs were from sex chromosomes.

### Allele Frequencies Calculations and Annotation

The allele frequencies were calculated for AB using PLINK’s *freq* option, resulting in. *frq* file in addition to PLINK’s bed/bim/fam files. A custom parser for gnomAD’s (Lek et al., 2016) vcf files (version hg19, downloaded from gnomad.broadinstitute.org) extracted allele frequencies for all available populations at all biallelic loci. Moreover, the script extracted the overall allele frequencies from the Greater Middle East (GME) Variome project (Scott et al., 2016). The script joins the three tables for those loci that agree in chromosome, position, reference, and alternative allele. We calculated the Z-score for the UAE allele frequencies with respect to allele frequencies of gnomAD populations, indicating significantly different allele frequencies. Subsequently, the table combining all allele frequency related data is converted into Variant Call Format (VCF) file. Further, we filter variants from the VCF file using the SQL based selection algebra of Variation Association Tools (VAT) (Wang et al., 2014) and then perform functional annotation using the variants annotation tool SnpEff (Cingolani et al., 2012).

### Admixture Calculation and Phylogenetic Analysis

In order to determine admixture, we used the tool ADMIXTURE (Alexander et al., 2009) in both unsupervised and supervised modes. For the unsupervised analysis, which initially does not assume any background information of number and allele frequencies of ancestral populations, the tool was run repeatedly with increasing values of *K* from 2 to 10, so as to show the cross-validation errors (see Supplementary Figure S1). The minimum value of this curve indicates the most likely number of ancestral populations (Alexander et al., 2009).

For the supervised admixture determination, eight continental populations from the HGDP: Middle East (comprising Druze, Bedouin and Palestinian), Central/South Asia, Sub-Saharan Africa, North Africa, Oceania, Native-America, East Asia, and Europe were used. The command “admixture*–supervised uae_hgdpLD.bed 8*” in ADMIXTURE’s supervised mode was used to predict the eight components of the abovementioned continental populations in all Emirati samples.

Additionally, phylogenetic analysis has been conducted to contextualize the UAE genomes in comparison to other world populations. This is achieved by calculating the intergenomic distance between a subset of 104 UAE individuals—for visualization purposes—of the UAE samples and the HGDP samples. We use the identity by state (IBS) distance measure from PLINK to calculate the mutual intergenome distances. The resulting distance matrix was subjected to neighbor joining and the phylogenetic tree was visualized using iToL (Letunic and Bork, 2011).

### Hierarchical Clustering (UPGMA) of Individuals

A distance matrix of all pairwise IBS dissimilarities (*1-ibs*) for samples was generated using PLINK’s distance parameter. The distance matrix was then subjected to hierarchical clustering with average linkage [also referred to as unweighted pair group method with arithmetic mean (UPGMA)] using scipy (Aivazis and Aivazis, 2011), yielding a dendrogram for all individuals.

### Principal Component Analysis (PCA)

Principal component analysis (PCA) was run on the previously described dataset ABH using the PLINK’s *pca* command. This produced output files with the first 20 ordered eigenvectors and eigenvalues, and the first two components for subsequent analysis and visualization were used.

### Admixture Informed PCA Plots

A Python program that generates novel, feature rich visualizations for genotyping data was developed. It parses output from PLINK (PCA results), ADMIXTURE (supervised admixture components), and geographic information for HGDP context samples. Based on matplotlib, the script plotPCA.py was used to generate a PCA plot, but in addition to conventional uni-colored dot plots of two to three components, each individual sample also contained admixture information in form of a pie chart, i.e., with pie-sections proportional to their respective components that contribute to a sample’s genotype. We further developed PCA plots with only selected populations (Europe, Middle East, Central/South-Asia, and Sub-Sahara) that were deemed most impactful based on the ADMIXTURE calculation of K with minimal cross validation error. For those PCA plots, we down-sample each population to 100 samples.

The visualization software is available on https://github.com/henschellab/populapy.

### Dendrogram Ordered Admixture Bar Plots

The plotting of admixture bar plots is conventionally arranged either by preconceived groupings or by lexicographic ordering of the individual admixture components. In contrast, the bar plots were arranged here according to the dendrogram derived from hierarchical clustering. This way, the genetic makeup of clusters becomes immediately apparent, as genetic similarity (as measured through 1-*ibs* dissimilarity) of samples translates into dendrogram proximity. Further, for comparison purposes, the dendrogram was consistently colored according to population membership of samples. Again, the Python scripts have been made available in the abovementioned repository.

### Genetic Diversity Calculation

#### Population Stratification and Population Cluster Analysis

We use PLINK (plink–cluster–genome) to perform population stratification. The first step produces a 2043 × 2043 distance matrix containing all pairwise IBS (1-*ibs*) distances of UAE and HGDP samples. We then use Pandas and Python to calculate the average within-population distance (also referred to as population diversity or heterogeneity) and among-population distances (separation) for all populations. Formally, the within-population distance is given by the average of all distances of pairs of an individual in a population. The pairwise separation between two populations is calculated by averaging over all pairs with one being a member in one population and the other being member in the other population. The separation of one population is then the average of separation values to all other populations. For the sake of an unbiased separation value for the UAE, we omit HGDP Middle East. We then calculate compactness of a population as a measure for how well it clusters, by dividing heterogeneity with separation. Compactness can be interpreted as a measure of population stratification. We down-sample populations by randomly selecting 100 individuals per population (except for small populations like Oceania and North Africa). This process is repeated 100 times to estimate variance in separation, heterogeneity, and compactness. The (pairwise) separation values yield a new 8 × 8 distance matrix (seven HGDP populations plus the UAE) with pairwise distances between populations, to which we apply hierarchical clustering with average linkage. In addition, for each population, we calculate heterogeneity (average within-population distance), separation (average among-population distance), and the separation/heterogeneity ratio, which balances within-cluster homogeneity against between-cluster separation. We perform the same steps for six UAE Emirates separately, with cluster labeling derived from the samples’ respective birth places. We finally combine all HGDP populations and individual Emirates, to put inter-Emirate differences into global perspective. The Jupyter notebook that reproducibly details all steps and calculations is provided in the abovementioned repository.

#### F-Statistics

We calculate mean Weir-Cockerham F_ST_ values using vcftools version 0.1.16 (Danecek et al., 2011). For each population pair, we issue commands of the form

vcftools--vcfuae_hgdp.vcf--weir-fst-pop<pop1>.txt

--weir-fst-pop<pop1>.txt--outpairFst_<pop1>_<pop2>

where uae_hgdp.vcf is the vcf version of the dataset ABH, described in Section “Genotype Array Data Pre-Processing and Merging of Several 188 Genotyping Datasets.”

#### AMOVA Analysis

The analysis of molecular variance (AMOVA) is used to estimate the UAE population differentiation using molecular markers from the seven UAE subpopulations (grouped by their birth place). The analysis was conducted using the Arlequin (Excoffier et al., 2007) command line tool (arlecore). Three runs of the AMOVA analysis were performed based in different grouping of the UAE and five HGDP world populations excluding (Americans, Oceanians, and East Asians). The AMOVA analysis calculates how much of this differentiation is due to differences between populations, between individuals within populations, and within individuals.

## Results

### Global Mapping and Admixture of the UAE Population

**FIGURE 1:**
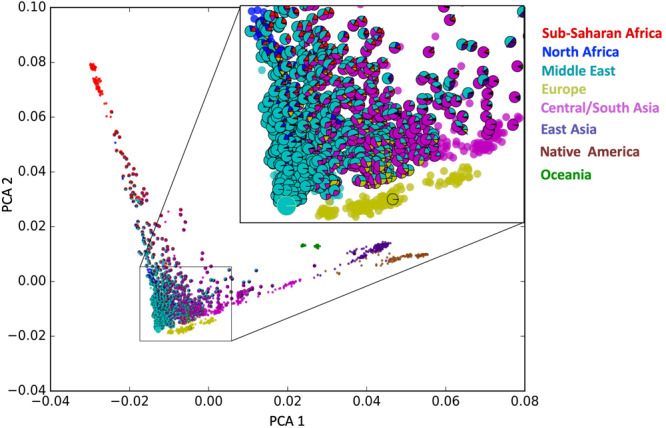
Admixture informed principal component analysis (PCA) plot. Each outlined sample denoting an individual studied here is represented as a pie chart of ancestral composition as determined with the Admixture software tool in supervised mode. In addition, HGDP samples from model populations are shown as circles in their respective colors (no outline).

The genotype array analysis yielded the allele frequencies for 2,382,081 exonic loci after the quality control step. In the PCA that is Figure 1, the HGDP samples span along two major internal axes, one which is predominantly Asian/American, and the second being predominantly African, an observation consistent with previous studies (Lu and Xu, 2013; Haber et al., 2016).

In Figure 1, the Emirati population (samples with outline) maps to this PCA plot where the internal axes intersect. In contrast to the HGDP populations which cluster in a relatively tight group, the UAE subjects fill the entire spectrum, loosely around the intersection of those two axes. This loose clustering suggests a continuous distribution of samples in the apex of the internal axes. Each UAE subject is represented with a pie chart (outlined) showing the ancestral composition of the individuals as determined by the ADMIXTURE software tool (see section “Admixture Calculation and Phylogenetic Analysis”). The pie charts largely correspond to their mapping on the principal components.

Additional PCA has been performed by removing the continental populations that have only marginal contribution to the ancestry of UAE citizens (i.e., Americans, Oceanians, and East Asians) (Supplementary Figure S2). Those plots are based on down-sampled population sizes and corroborate the general picture provided by the overall PCA: a widespread of UAE samples, high diversity, low stratification, and compactness.

**FIGURE 2:**
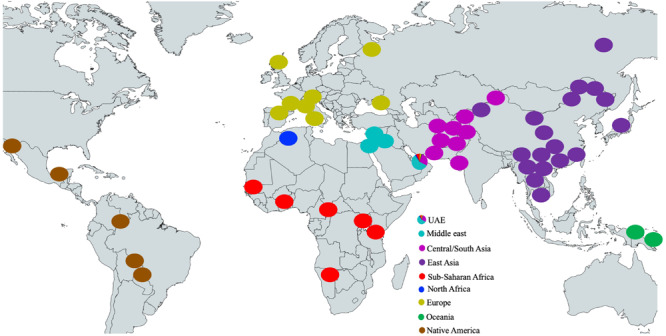
Locations of the HGDP model populations and the accumulated ancestral population proportions for the UAE population (shown as a pie chart). The basic(grey) world map is created with mapchart.net.

In Figure 2, the locations of the HGDP model populations are shown as circles colored according to their respective populations as well as the accumulated ancestral population proportions for the UAE population (shown as a pie chart representing the UAE). Unsurprisingly, the Middle Eastern component is dominant within the UAE population. The influence of ethnic groups from regions that are juxtaposed to the Arabian Peninsula, specifically from Central/South Asia, Sub-Saharan Africa, and to a lesser extent Europe and North Africa on the contemporary gene pool of the UAE is also not surprising, considering the interaction between populations over millennia.

**FIGURE 3:**
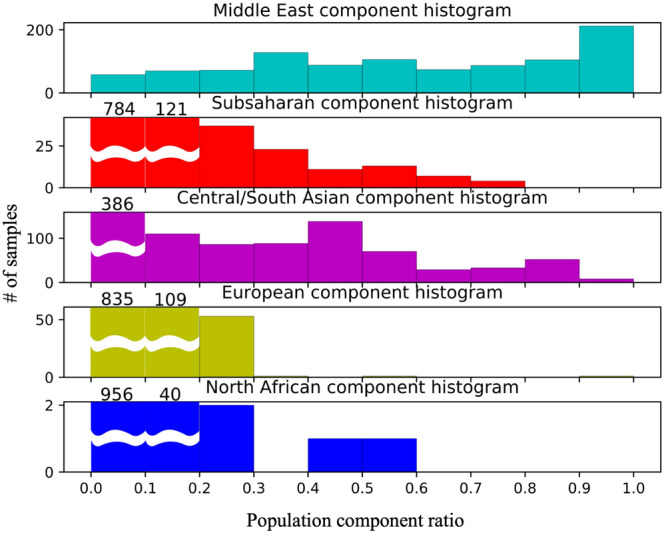
Component histograms for African, Middle Eastern, and Central/South Asian contributions in 1000 Emiratis. All levels of African and Middle Eastern contributions are observed.

Approximately 10% of the samples have >99% of their genotype assigned to the Middle Eastern model population from HGDP (see Figure 2 UAE pie chart). However, in contrast to previous studies in the region (Qatar, Kuwait) (Omberg et al., 2012; Alsmadi et al., 2014; John et al., 2015; Thareja et al., 2015; Fakhro et al., 2016), the remaining individuals do not fall clearly into either category with one single dominating component. Instead, a gradient continuum was observed for the three major ancestral components, as evidenced by the component histograms in Figure 3. Again, it should also be noted that the numbers of samples and genotypes in this UAE study are larger and thus less sensitive to falsely detecting clusters that in reality are a result of under-sampling.

### Admixture and Phylogenetic Analysis

The admixture cross validation for different numbers of ancestral populations yielded a minimal error for K = 5 (see Supplementary Figure S1). We use this K to guide the generation of PCA plots with limited numbers of populations.

**FIGURE 4:**
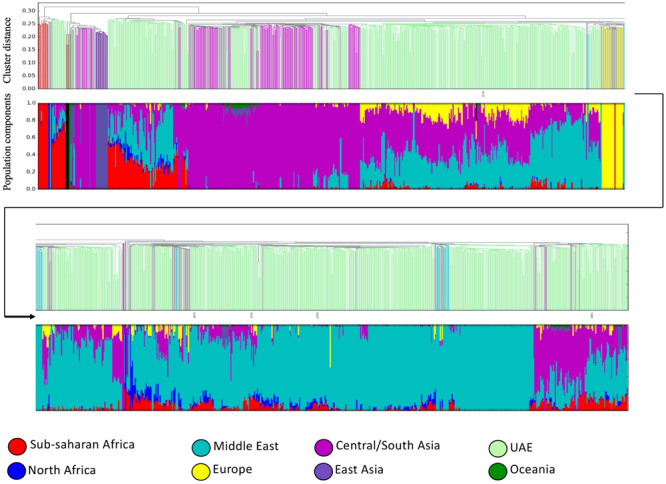
Dendrogram ordered admixture barplots. Ancestries and population structuring of UAE individuals revealed by a supervised admixture analysis against eight ancestral world populations.

**FIGURE 5:**
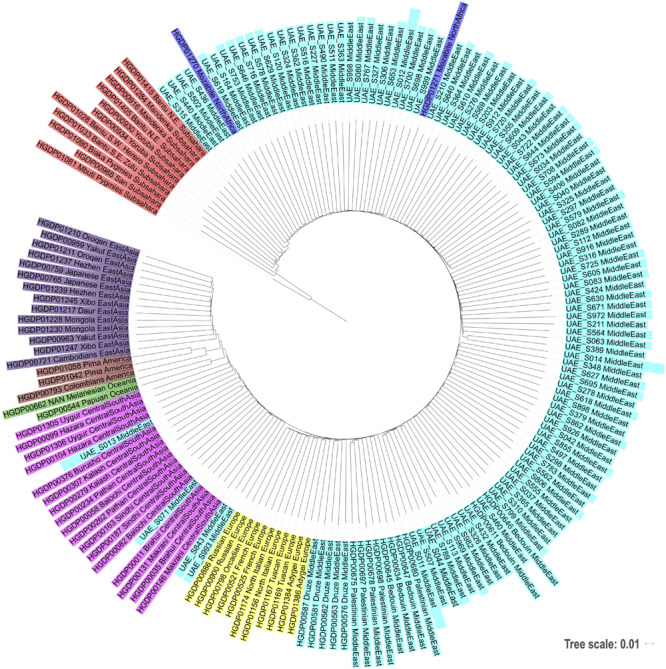
Phylogenetic tree based on neighbor joining and identity by state distances contextualizing the local samples of this study against world populations.

The bar plot generated by the ADMIXTURE Software tool shown in Figure 4 was generated and ordered according to the arrangement of leaves in the dendrogram from hierarchical clustering. The phylogenetic tree (Figure 5) shows relatively concordant clustering of individuals with similar ethnic ratios, except for a number of outliers. As eluded to, the North African contribution, using the HGDP cohort, to the Emirati gene pool is very limited (Figure 4). Even those UAE samples that appear next to the North African samples in the admixture/PCA plot in Figure 1 are often composed of different major components, an aspect that would not have been inferable from conventional PCA plots. Interestingly, those with North African contributions are dispersed with individuals who have a predominance of Middle Eastern contribution (see Figure 5). There were also four individuals identified as Middle Eastern, that are dispersed with the Central/South Asian cluster. As expected, native American, Oceanian, and Far Eastern contributions are very limited as evident in Figure 4.

Given this more comprehensive picture of the subpopulations and in order to present the diversity within the population of the UAE, four individuals were selected from distinct parts of the PCA plot for WGS. These four individuals were centrally located within their respective population groups in the PCA plot. Representatives with predominantly Middle Eastern (UAE S011) and Central/South Asian ancestry (UAE S013) were selected. The remaining two representatives (UAE S012 and UAE S014) had different admixture ratios with influences from Sub-Saharan Africa, North Africa, Middle East, Central/South Asia, and Europe. The influence from Oceania, native Americans, and East Asia was minimal (no more than 1.08%) in all four representatives. WGS for these four individuals has been completed and the results of their analysis are unpublished.

### Genetic Diversity Analysis

#### AMOVA Analysis

The comparison of the UAE seven subpopulations (grouped by their birthplace in the emirates), using AMOVA revealed that only 0.24400% of the total variation was found among subpopulations (individual’s birthplaces) while the rest (96%) was within subpopulations’ individuals scoring a fixation index (Fst) value of (0.00244) (see Table 1). This shows a strong within individual’s diversity in comparison to the diversity among the populations.

**TABLE 1:** Global AMOVA results as a weighted average over loci.

Populations	Source of variation	d.f*	Sum of squares	Variance components	Percentage variation	Fixation index (Fst)
UAE’s subpopulations	Among population	6	324675.507	86.21332	0.244	0.00244
	Among individuals within population	763	28700974.7	980.57196	2.77519	
	Within Individuals	800	27387119	34266.7071	96.98081	

The analysis of the UAE subpopulations grouped by seven different birthplaces. d.f* = degrees of freedoms.

Additionally, AMOVA analysis based on different grouping of the 800 individuals in seven UAE subpopulations and 663 individuals from five HGDP world populations—(*a*) 12 groups (UAE seven subpopulations and five HGDP five populations, (*b*) six groups (UAE and five HGDP), and (*c*) two groups (UAE and HGDP) has shown higher “among population” diversity in the populations structures (*a*) and (*c*) (Supplementary Table S1). The population pairwise genetic distances for the above population structures are generated using the Slatkin (Excoffier and Slatkin, 1995) distance measure is shown in Supplementary Figure S3.

#### Clustering

**FIGURE 6:**
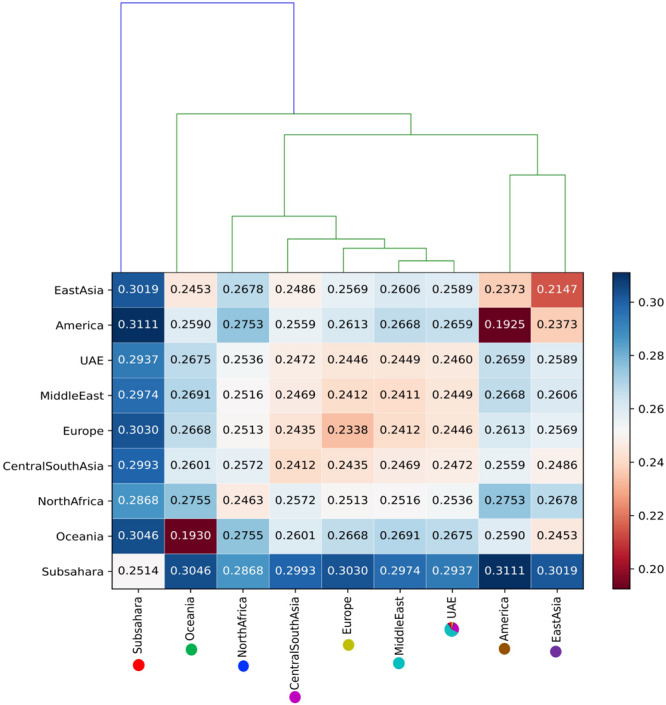
The color-coded distance matrix, arranged by the clustering dendrogram between the UAE population and against eight ancestral world populations.

Identity by state-based pairwise distances between all samples showed that the UAE samples have a comparatively high heterogeneity. Table 2 shows cluster characteristics of the UAE in comparison to (down-sampled) HGDP populations. Remarkably, the UAE has the lowest separation/heterogeneity (compactness) ratio, indicating that UAE samples do not form a compact cluster (for example, Americans or Oceanians) when contextualized with HGDP populations (Table 2). This is due to comparatively high heterogeneity (e.g., higher than Europe, East Asia, America, and Oceania, comparable to Central South Asia, North Africa, and the Middle East) while also exhibiting comparatively low separation to other populations. The color-coded population distance matrix, arranged by the clustering dendrogram, is shown in Figure 6. As expected, the UAE is closest to HGDP’s Middle East, while the remaining world populations arrange in line with the HGDP. Interestingly, in terms of separation, UAE is most distant from the Sub-Saharan Africa, even though a substantial Sub-Saharan African component has been revealed in the admixture analysis in UAE nationals. We attribute this to the high diversity in Africans being the dominant factor. Hierarchical clustering for UAE birthplaces mirrors geographical proximity and historic relations (Supplementary Figure S4). However, distances are very small, as can be seen when contextualizing the UAE national birthplaces in a global context (Supplementary Figure S5).

**TABLE 2:** Cluster characteristics of the UAE in comparison to (down-sampled) HGDP populations.

	Compactness mean	Compact std	Heterogenity mean	Separation mean	*T*	*P*
UAE	1.063692236	0.00339496	0.2459575	0.261619731	0	1
Central South Asia	1.073312712	0.00168414	0.24111312	0.258789099	−25.25837531	7.64E-64
North Africa	1.083155129	0.0003187	0.24629228	0.266772749	−56.79171903	1.68E-124
Europe	1.11681804	0.00101625	0.23375831	0.261065394	−149.1605526	2.29E-205
Sub-Saharan Africa	1.19385538	0.0027968	0.25134665	0.300070385	−294.4347462	1.46E-263
East Asia	1.208236732	0.0025765	0.21478897	0.259514893	−337.4518228	2.90E-275
America	1.384473213	0.00336748	0.19251678	0.266533055	−667.4734845	0
Oceania	1.390620317	0.00047624	0.19300375	0.268394939	−948.8630385	0

*The IBS-based pairwise distances between all samples showing that the UAE samples have a comparatively high heterogeneity.*

#### F-Statistics

**FIGURE 7:**
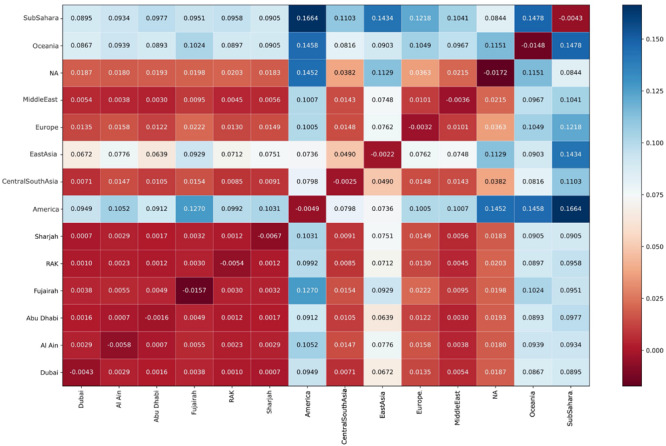
Color coded Mean Weir-Cockerham F_ST_ values. UAE F_ST_ values are closest to Central/South-Asia, Middle East, Europe, and North Africa. All Emirates exhibit very low F_ST_ values amongst each other in comparison to global F_ST_ values.

The color coded F_ST_ values are shown in Figure 7. As expected, F_ST_ values are closest to Central/South-Asia, Middle East, Europe, and North Africa, whereas remarkably, all Emirates exhibit very low F_ST_ values amongst each other in comparison to global F_ST_ values.

### UAE Specific Allele Frequencies

We identified 46,481 variant loci with significantly different allele frequencies (|Z| > 4, corresponding to a *p*-value of 6.3 × 10^–5^) when compared to allele frequencies reported for gnomAD and the GME populations. Nine hundred and six of these were annotated by the following high impact SnpEff functional assignments: Non-synonymous Coding (880), Start-Gained (six), Stop Gained (five), and Splice Site Region (15), as shown in Table 3.

**TABLE 3:** Significantly different allele frequencies and break down by SnpEff effect category.

Z-score	Total	Non-synonymous coding	Start gained	Stop gained	Exon	Intron	Splice-site region	Others
Z < -4	1261	6	0	0	6	523	0	726
Z > 4	45,220	874	6	5	305	18,649	15	25,366
| Z| > 4	46,481	880	6	5	311	19,172	15	26,092

*The SnpEff categories Intergenic, Upstream, Downstream, Synonymous coding, 3′-UTR, and 5′-UTR are combined in the column “Others.”*

**FIGURE 8:**
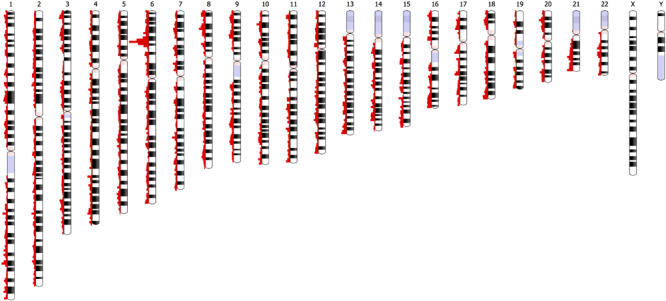
Depiction of the chromosomal locations of the 46,481 variants with significantly different UAE specific allele frequencies (|Z| > 4, *p*-value = 6.3 × 10^– 5^) relative to a comparison to frequencies of the gnomAD populations.

The distribution of the 46,481 variant loci throughout the genome is depicted in Figure 8. The histograms show the number of variants at specific chromosomal locations. There are peaks and troughs in variant numbers throughout all 22 chromosomes. As expected, there is a significant spike in the number of variants on the short arm of chromosome 6. This region around 6p21.3 is known as the human major histocompatibility complex (MHC), a region that is known to contain highly polymorphic genes, many of which are duplicated (Dawkins et al., 1999). There is no comparative data available for the short arms of the five acrocentric chromosomes (13, 14, 15, 21, and 22) in which the centromere is located quite near one end of the chromosomes, and in which there are no unique genes, or the genome remains uncharacterized.

Furthermore, no comparisons were available for the heterochromatic centromeric regions of chromosomes 1, 9, and 16, regions that are known to be highly unstable.

## Discussion

This comprehensive, large scale population stratification study of the population of the UAE reveals a detailed structure of the populations that inhabit the south-eastern corner of the Arabian Peninsula. Specifically, the study reveals a continuous genotype landscape, characterized by gradual admixture, without the formation of the traditional clusters in the mathematical sense. This is due to low separation values of the UAE with regard to world populations. The same effect holds true for individual Emirates, exhibiting within-population differences nearly as high as among-population differences (yielding compactness values close to 1.0). Had there been clear distinct clusters, it would reflect in higher cluster coefficients with high separation and low within-cluster heterogeneity—for example, in Oceania or Native America (both 1.38). The impact of respective Middle Eastern, Central/South Asian, and African influences can be appreciated in Figure 1. Others have proposed the temporal concept of a continuous genotype landscape in the Middle East populations. For example, Haber et al. (2017) sequenced and compared five ancient ∼37,000-year-old individuals from the city of Sidon and 99 individuals from present-day Lebanon. The study describes a continuum of the Canaanite population, an ethnic group of the Levant region, from the coast to inland populations of what is now Jordan. The Canaanite gene pool is said to have arisen from admixture between the local Neolithic populations and immigrants from the eastern extremes of the Middle East, in what is present-day Iran.

Nevertheless, the gradual admixture that was observed here in the UAE study was enhanced by a novel visualization tool, which graphically combines admixture information in the form of a pie chart with PCA. The use of both PCA and admixture analyses is commonly used, for example, in the Indian study showing the genetic influence from Africa (Narang et al., 2011) and Figure 1 combines these two traditionally separate functions. The format of the figure also alleviates potential projection bias in that the pie charts retain presents information on the extent of admixture and the populations involved, in addition to the first two principal components.

The general results compare well to previous studies from the region, specifically from Qatar (Omberg et al., 2012; Fakhro et al., 2016) and Kuwait (Alsmadi et al., 2014; John et al., 2015), which revealed overall concordance as all three populations show a diverse, admixed population, positioned similarly with respect to other world populations. Furthermore, previous analyses of the populations of the Arabian Peninsula by Hunter-Zinck et al. (2010) and Alsmadi et al. (2013) found three distinct primary ancestral groups, the Bedouins, the Persians from Central and South Asia, and Africans (Omberg et al., 2012). The predominant ancestral pool is the Bedouins from the Middle East (Figure 2), the people that originated from the population that was formed in the Arabian peninsula. However, this study does not confirm the notion of three clusters in the mathematical sense as seen in the other studies. Instead, a continuous spectrum of genotype distribution was observed (Figures 1, 4). An example in Figure 1 shows a slight gap below the one UAE individual with strong influence from Sub-Saharan Africa. A conventional PCA would have resulted in the misleading conclusion that only samples above the gap constitute a cluster. However, as the admixture pie charts in the plot reveal, the samples below the gap have a strong Sub-Saharan African component. We argue therefore that clustering in this type of analysis is more likely a result of under-sampling, rather than true stratification. This finding is corroborated by continuous gradients of ancestral population contributions, indicating that admixture along the Eastern seaboard and south eastern tip of the Arabian Peninsula occurred gradually and without strong social stratification borders. The UAE is a relatively young nation, formed by a federation comprising seven emirates on the 2 December 1971. The diversity seen in the contemporary UAE population arose over generations before the current political borders were drawn. To confirm this, the study also investigated admixture with regard to birth location throughout the UAE. Samples studied here were collected from all seven emirates. There was no clear correlation between birth location throughout the UAE and ancestral background.

We only observed very subtle stratification effects and admixture, for example, individuals with substantial Sub-Saharan component were born in nearly all the seven Emirates of the UAE. This is found to be in agreement with the preformed AMOVA analysis results (Table 1).

The diversity observed in the contemporary UAE population could have arisen in two possible ways. The generally held understanding of the peopling of the world has origins in Sub-Saharan Africa, migrating northward, crossing into the Arabian Peninsula, and radiating eastward throughout Asia, across the Bering Strait which now separates Russia from Alaska, followed by the colonization of North and subsequently the South American continent (Nielsen et al., 2017). The current population landscape in the UAE could have arisen from the contribution from ancestral populations who were already diverse at the time of immigration out of Africa. The oldest, and arguably the most diverse populations would reside in North Africa, the Middle East (the Arabian Peninsula and Levantine areas), Central/South Asia, two of which are adjacent to Sub-Saharan Africa. The three most dominant genetic contributions to the UAE (Figure 3) and minor contributions come from these four regions (Figure 4).

Alternatively, the dispersed distribution of the UAE subjects, when considered in concert with the ancestral composition, shows that the contemporary population of the country was historically influenced by populations from surrounding regions (Figure 5). It is widely believed modern humans migrating from Africa did so some 85,000 years ago (Cann et al., 1987; Forster, 2004; Kundu and Ghosh, 2015) traveling along the southern coast of Asia via the Arabian Peninsula. In more recent times, the Bedouins, a population that dates back to at least 850 BC rose to become the dominant group (Marx, 1967; Cavalli-Sforza et al., 1988; Mohammad et al., 2009; Hunter-Zinck et al., 2010). They are nomadic, desert-dwelling Arab peoples of the Middle East. Traditionally they lived in tents, moving with their herds across vast areas of arid land in search of grazing areas and water. They were also traders imposing taxation on trade caravans, collecting tributes from non-Bedouin settlements, and by transporting goods and people in caravans across routes that were familiar to them. As they moved throughout the Arabian Peninsula, their travels took them to non-Bedouin outpost adjacent to or within to the Levant region as well as Central Asia or Persian. According to the data presented here, interaction with North Africa occurred, but to a lesser extent (see Figure 1). Along the southern coast of the Arabian Peninsula including the area at the south eastern tip which includes the UAE, migration occurred across the Arabian Gulf into South Asia and where the Red Sea and the Gulf of Aden meet into Africa. The diversity is potentially a function of this migratory pattern when the peoples of these regions moved back and forth between the African and Asian continents. As mentioned, there are African influences is western parts of India (Narang et al., 2011). Furthermore, a recent genome-wide diversity assessment of the Markanis, a population that now resides on the Arabian Gulf coast in Pakistan has confirmed the interaction between Bantu-speakers from eastern or south-eastern Africa and the Balush tribes of Asia, that occurred as recently as sea trade dominated by the Omani Empire (Laso-Jadart et al., 2017). The pattern of admixture is influenced by what is best described as a “merry-go-round” effect, in which the Bedouin people would have interacted with people from neighboring populations as they dropped in and out of settlements within and adjacent to the Arabian Peninsula. The dispersed nature of the population represented in the PCA plot that is Figure 1 suggests that admixture in the region happened over a larger period of time or alternatively, diversity was introduced already during primary immigration events and remained.

The Emirati population is thus less stratified than what would have been expected according to the Kuwaiti (Alsmadi et al., 2013, 2014; John et al., 2015; Thareja et al., 2015) and Qatari (Omberg et al., 2012; Fakhro et al., 2016) studies. It should be noted that the UAE study is comparatively larger and thus less prone to selection bias. It is also noteworthy that the North African contribution to the UAE gene pool is very limited, in contrast to influences from Central/South Asia and Sub-Saharan Africa. Those UAE samples that appear next to the North African samples in Figure 1 are often composed of different major components, an aspect that would not have been inferable from conventional PCA plots. Without the admixture ratios presented as pie-charts, those samples would have been considered North African like, and could encounter negative consequences, if subjected to diagnostics and/or treatment based on this miscategorization.

Another important result from the genotyping is the provision of allele frequencies for 2.3 million variant loci. Hitherto statistically underpowered, this study is a timely effort and a statistically significant estimation derived from over 1000 independent random samples. Note that databases such as ExAC and gnomAD (Lek et al., 2016) do not provide this type of information due to the lack of Arabian and UAE specific sampling efforts. The GME database provides accumulated allele frequencies for the “GME” only, a region that spans from the North of Africa from Morocco to South Asia to what is now Pakistan. As such, the GME dataset (Scott et al., 2016) comes from the influence of at least 19 ethnic groups that are known to live in this greater region. The study presented here recognizes that an appreciation of ethnicity specific differences may, in turn, provide insights into subpopulation specific disease associated variants, an effort that remains elusive.

By combining the statistical strength from array genotyping for 1000 samples and the high coverage of WGS for four representatives, the data in this study provide a rich base for future genomic studies in the UAE and in the Arabian Peninsula, which has yet to be studied. This analysis has established a reference data collection that has breadth and depth. The large set of comprehensive genotype arrays has not only facilitated the mapping of the local genetic landscape in a global context within the major world populations, it has also provided a rich set of country specific allele frequencies. On the other hand, the four genomes will work as a more suitable reference for the next “*n* + 1” local sequencing effort. Even though the population at hand is small, due to its diversity, the choice of a suitable reference is not a trivial undertaking. However, at least one of the genomes is likely a more adequate match than the conventional reference genomes (hg19, GRCh38) in terms of read mapping percentage and variant calling.

## Conclusion

In summary, efforts here show that affordable genotyping using arrays helps in generating a comprehensive population overview, which in turn provides guidance in choosing adequate representatives; in terms of their centrality in the subpopulation and their admixture content; for more in depth study (i.e., WGS). Of the 2.3 million variants identified, there were 46,481 variants (approximately 2%) with allele frequencies that were significantly different, when compared to other populations. These variants were relatively evenly distributed across all 22 chromosomes with the MHC on the short arm of chromosome 6 containing the dominant number of variations (Figure 8), as expected. The study demonstrates how genotype array data and WGS are complementary sources for anthropological studies, taking into account the underlying genetic structure of each individual.

## Data Availability Statement

The Data described in this article is deposited at the European Genome Archive (EGA) under the accession number EGAS00001004362.

## Ethics Statement

The studies involving human participants were reviewed and approved by Mafraq Hospital in Abu Dhabi, United Arab Emirates (UAE). The patients/participants provided their written informed consent to participate in this study.

## Author Contributions

HA obtained the funding for this study. HA and GT designed the study. GT, GD, and AH analyzed the data and prepared the manuscript. GT, HA, GD, and AH reviewed and edited the manuscript and contributed to the discussion.

## Conflict of Interest

The authors declare that the research was conducted in the absence of any commercial or financial relationships that could be construed as a potential conflict of interest.
