# Supplementary material for: Genetic Diversity and Low Stratification of the Population of the United Arab Emirates
Source: Front Genet. 2020 Jun 12;11:608. doi: 10.3389/fgene.2020.00608 (PMC7304494; doi:10.3389/fgene.2020.00608)
Supplement: Supplementary file 1 [file Data_Sheet_1.docx]

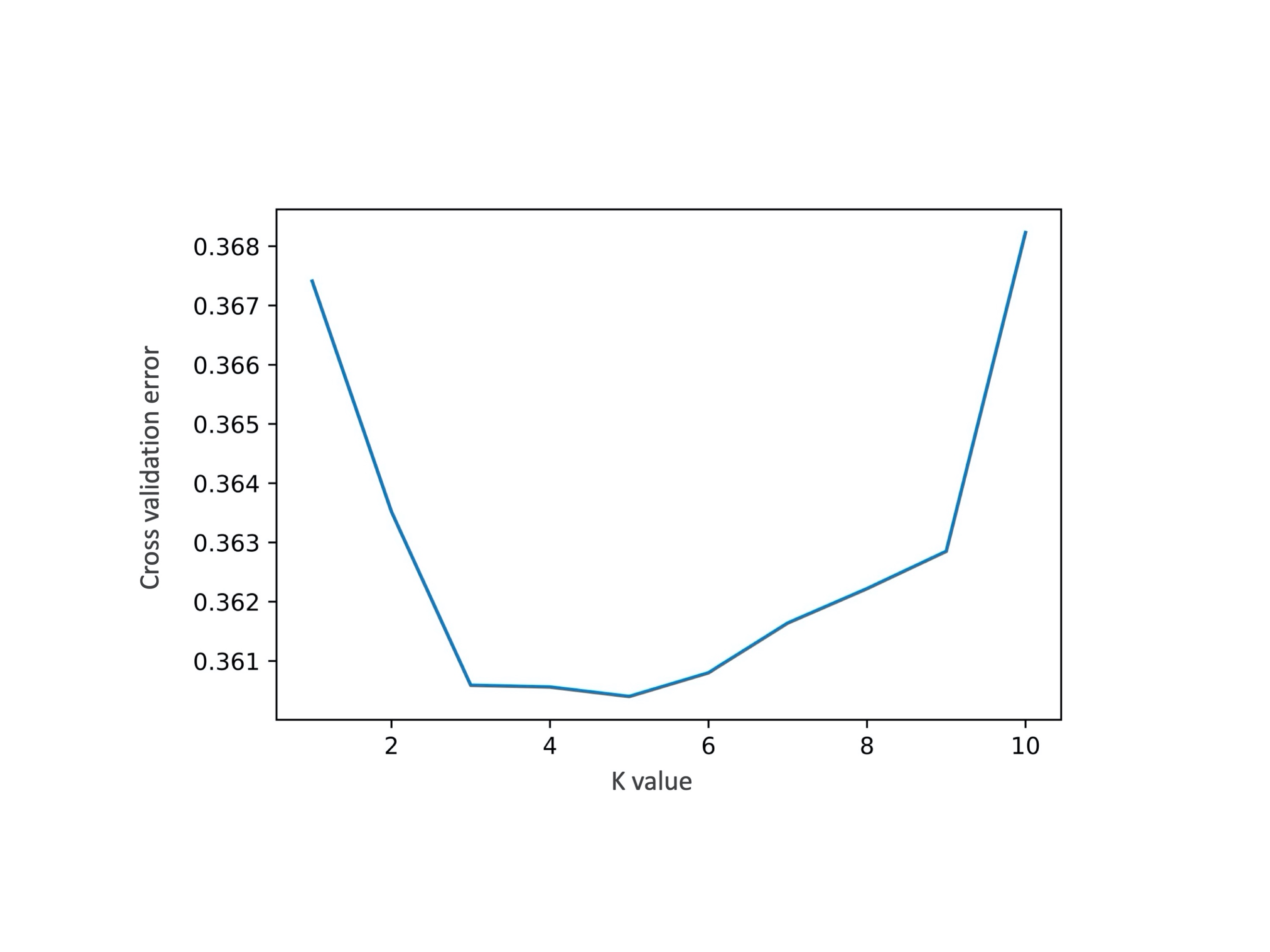


Figure S1: The admixture cross validation for different numbers of ancestral populations yielded a minimal error for K=5.


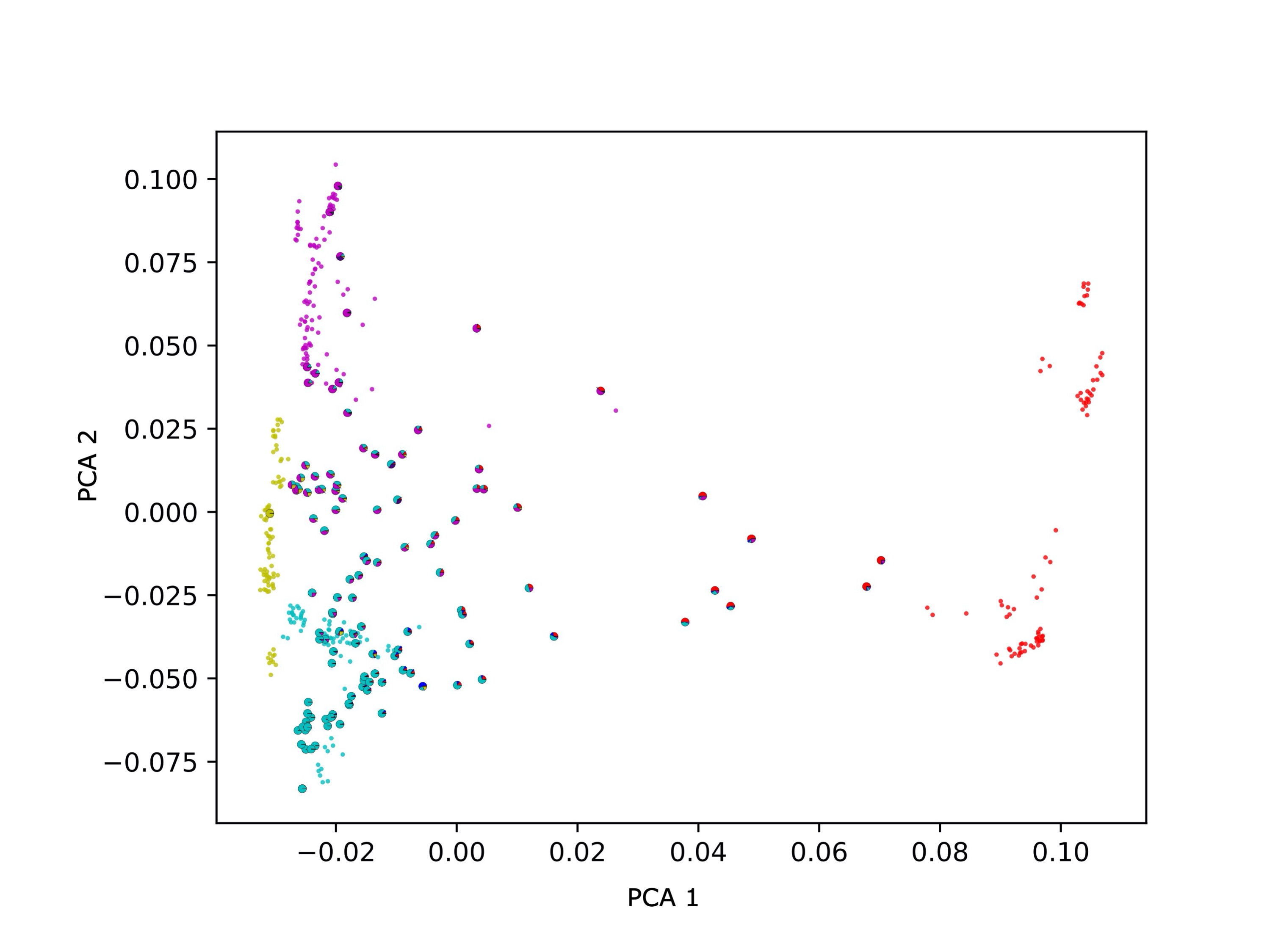


Figure S2: Based on down-sampled population sizes and corroborate the general picture provided by the overall PCA (Figure 1). The PCA is performed by removing of the continental populations that have only marginal contribution to the ancestry of UAE citizens (i.e. Americans, Oceanians and East Asians) showing a wide spread of UAE samples, high diversity, low stratification and compactness.

Table S1 Locus -by locus Analysis of molecular variance (AMOVA ) for 800 individuals in 7 UAE subpopulations and 663 individuals from 5 HGDP world populations. The analysis is based on different grouping of the studied populations a) 12 groups (UAE 7 subpopulations and 5 HGDP 5 populations, b) 2 groups (UAE and HGDP) and c) 6 groups (UAE and 5 HGDP).

| Populations | Source of variation | d.f | Sum of squares | Variance components | Percentage variation | Fixation index (Fst) |
| --- | --- | --- | --- | --- | --- | --- |
| UAE and HGDP  (12 grs) | Among populations | 11 | 3275119.89 | 1112.08413 | 3.07983 | 0.0308 |
|  | Among individuals within populations | 1451 | 52277272.832 | 1036.31947 | 2.87001 |  |
|  | Within Individuals | 1463 | 49677801.5 | 33960.21904 | 94.05016 |  |
| UAE (1 gr) and HGDP (1 gr) | Among population | 1 | 278072.299 | 165.67256 | 0.45938 | 0.00459 |
|  | Among individuals within population | 1461 | 55274320.421 | 1938.76210 | 5.37580 |  |
|  | Within Individuals | 1463 | 49677801.500 | 33960.21904 | 94.16483 |  |
| UAE (1 gr) and HGDP (5 grs) | Among population | 5 | 2950444.382 | 1451.33511 | 3.97788 | 0.03978 |
|  | Among individuals within population | 1457 | 52601948.338 | 1073.55105 | 2.94244 |  |
|  | Within Individuals | 1463 | 49677801.500 | 33960.21904 | 93.07968 |  |

d.f stands for degrees of freedoms

Figure S3: Population comparison using Slatkin’s distance to compute genetic distance derived from pairwise FST.

Figure S4: Hierarchical clustering for UAE birthplaces mirrors geographical proximity and historic relations.

Figure S5: Hierarchical clustering for UAE birthplaces and HGD populations.
