# Supplementary figures and images for: Genetic Diversity and Low Stratification of the Population of the United Arab Emirates
Source: Front Genet. 2020 Jun 12;11:608. doi: 10.3389/fgene.2020.00608 (PMC7304494; doi:10.3389/fgene.2020.00608)

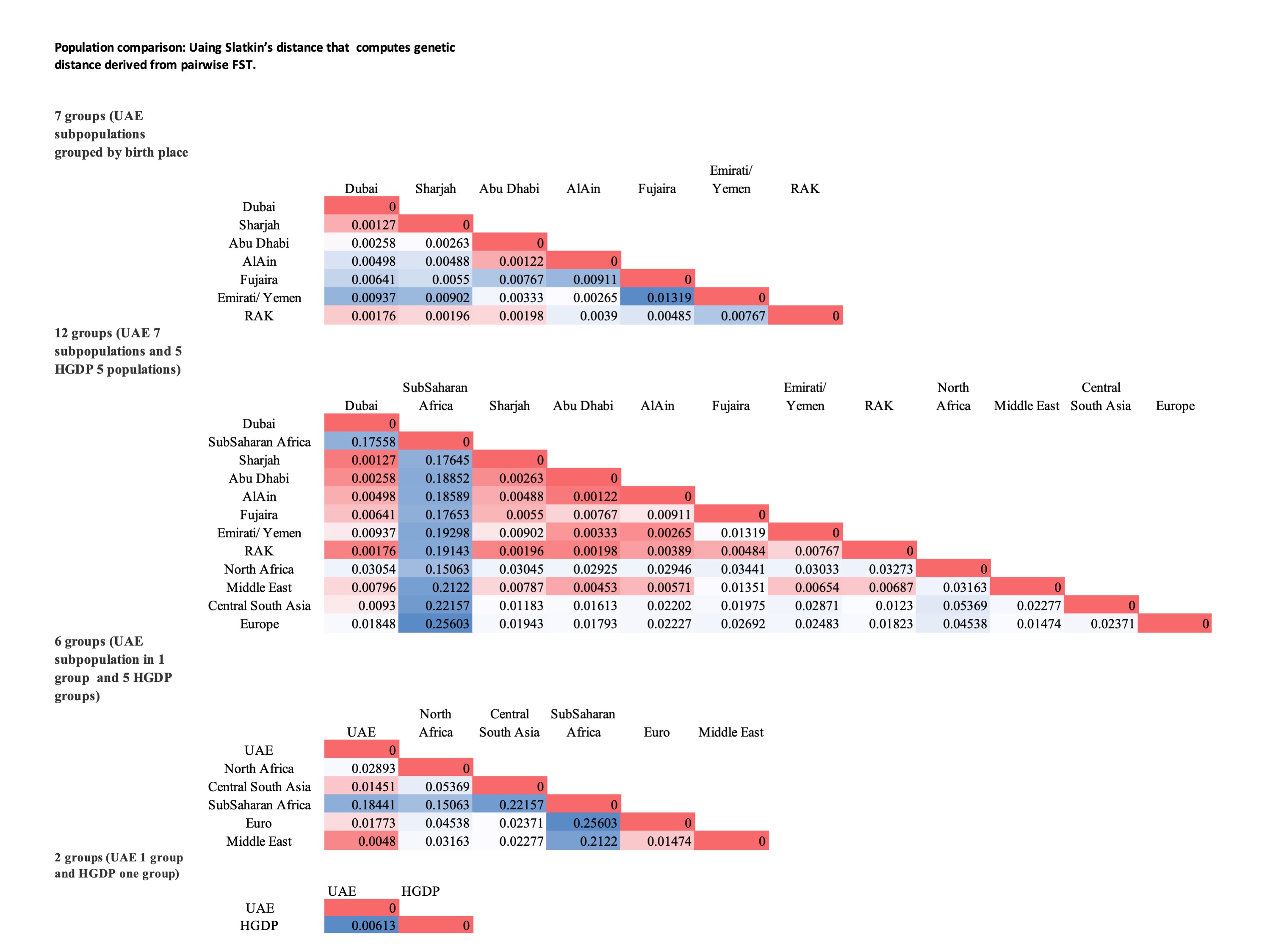

Supplement: Supplementary file 2 [file Image_1.JPEG]

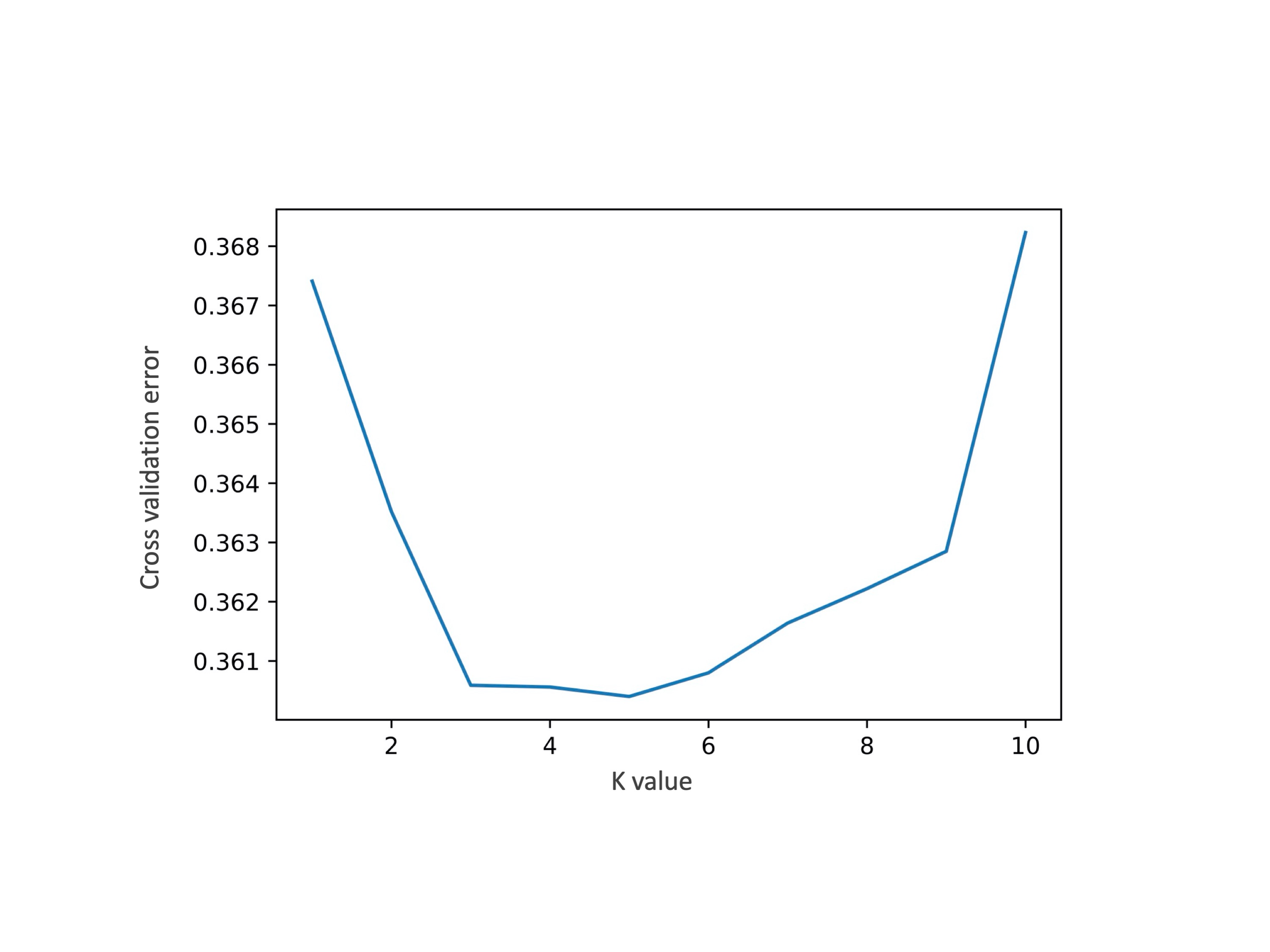

Supplement: Supplementary file 3 [file Image_2.JPEG]

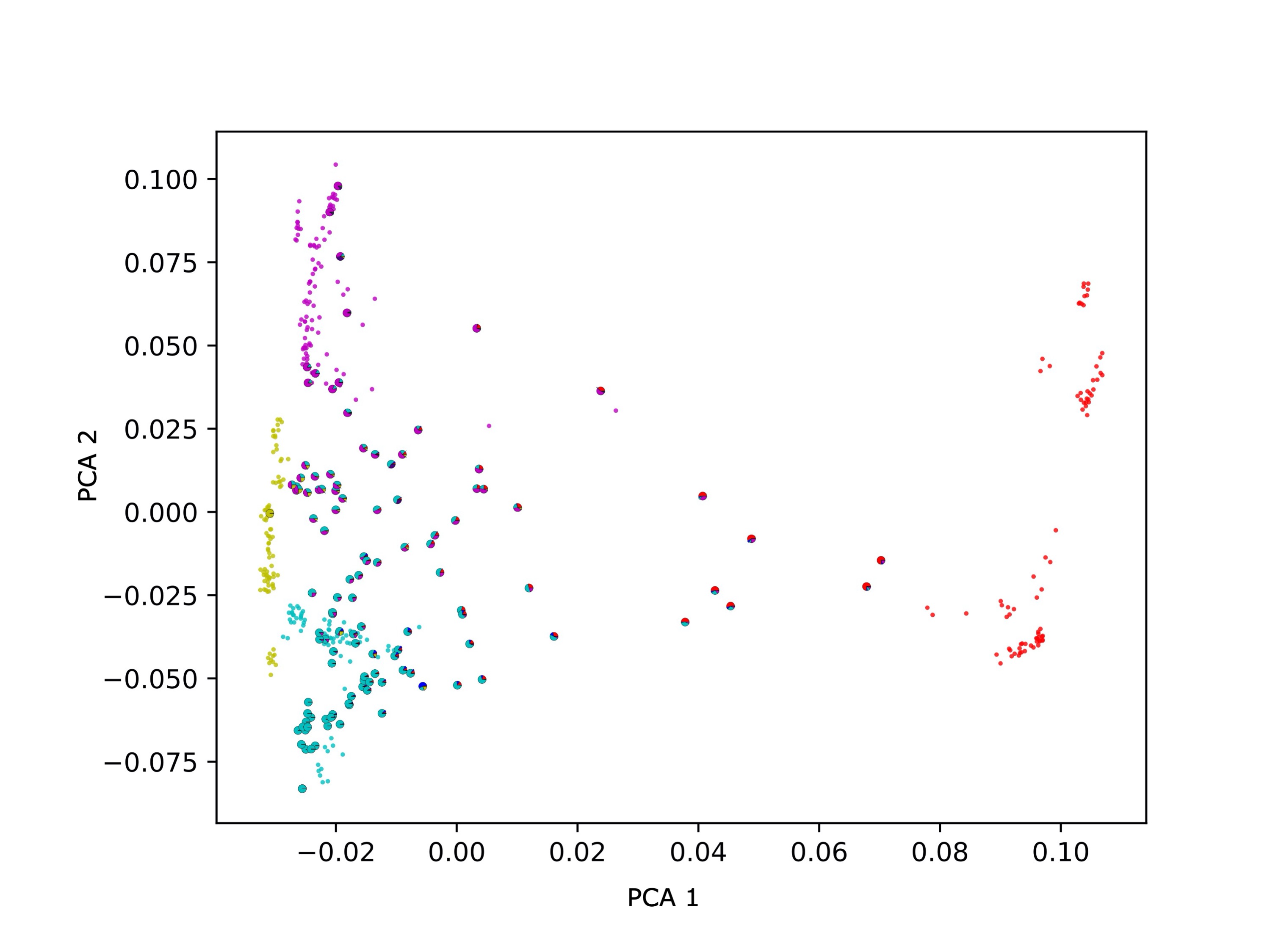

Supplement: Supplementary file 4 [file Image_3.JPEG]

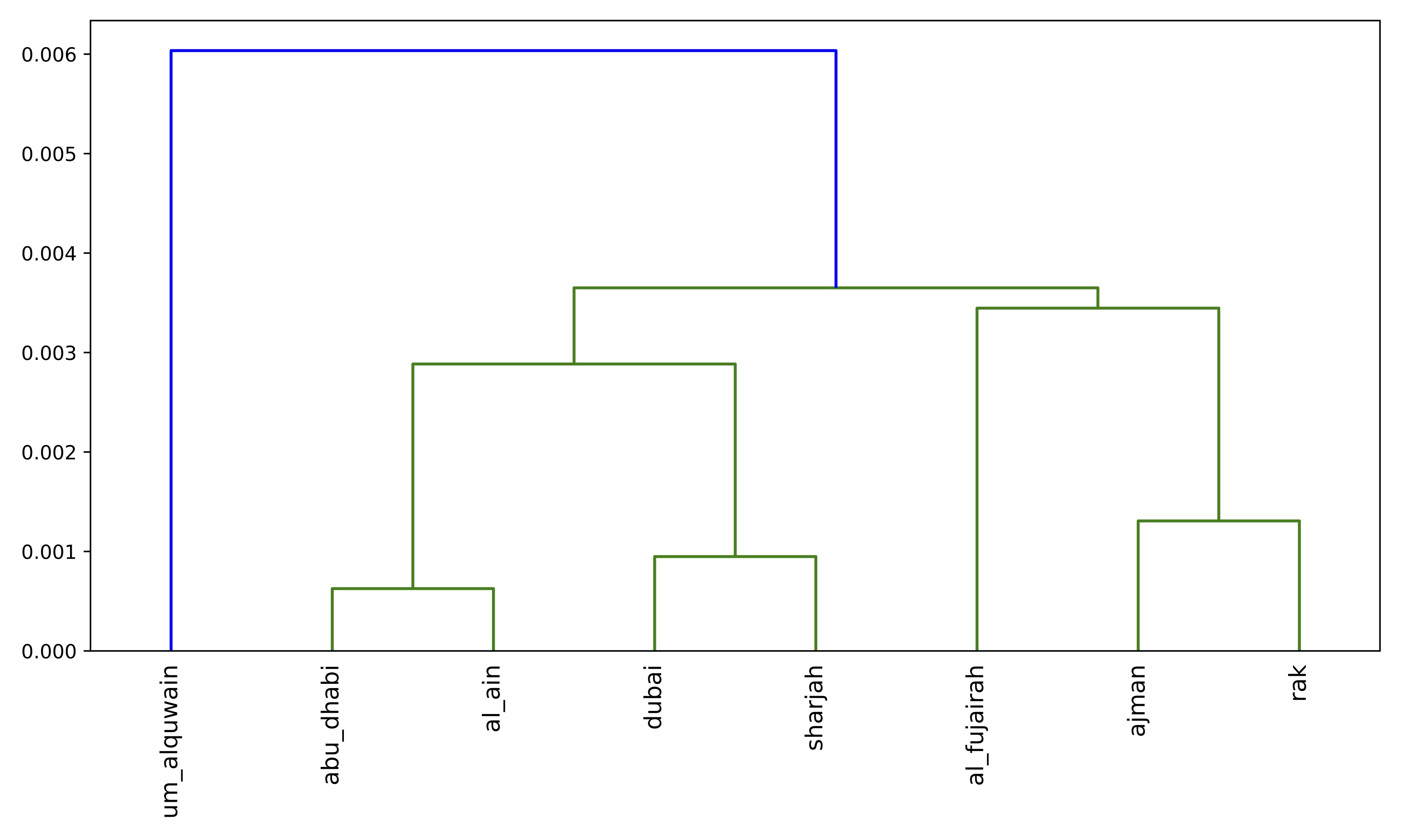

Supplement: Supplementary file 5 [file Image_4.JPEG]

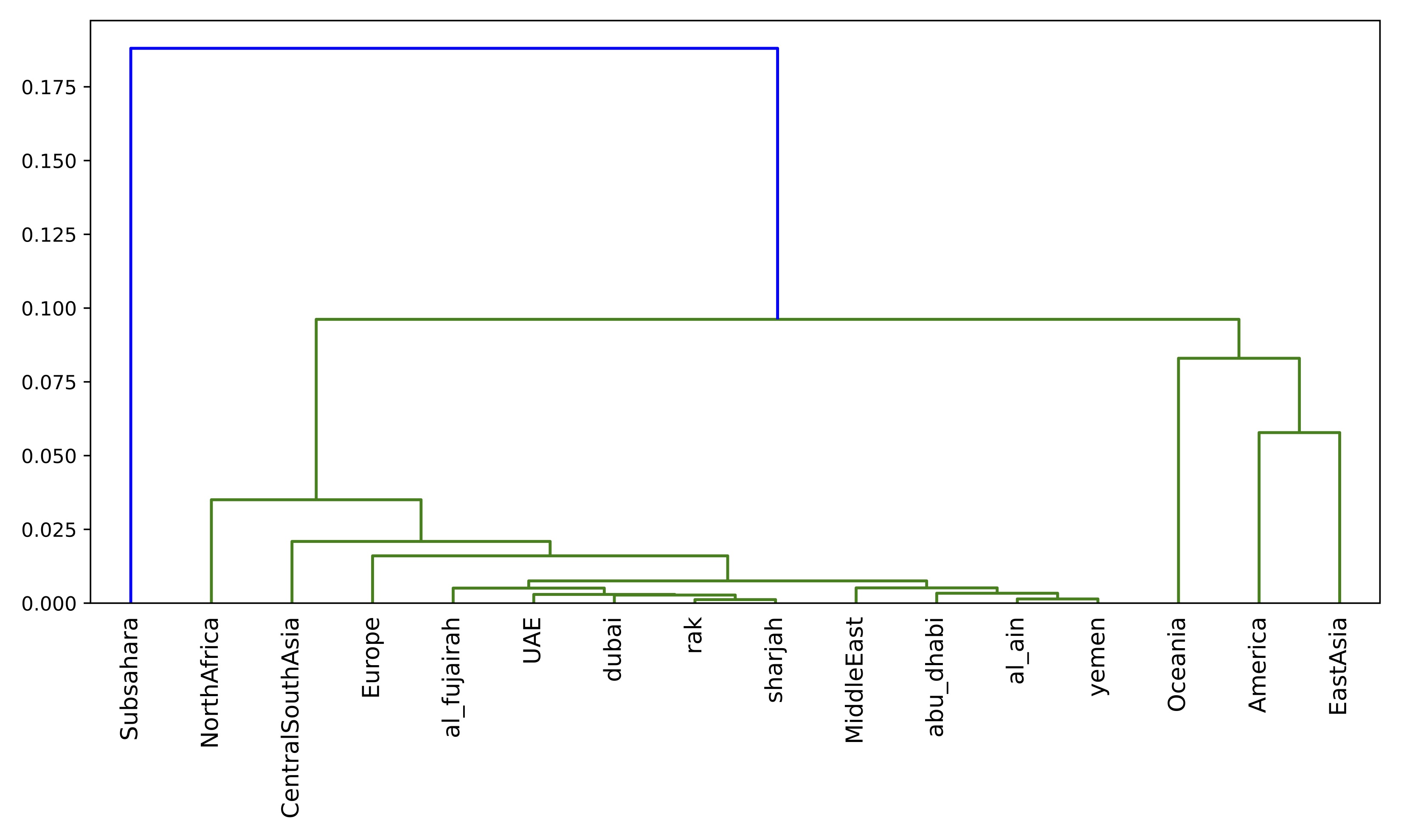

Supplement: Supplementary file 6 [file Image_5.JPEG]
